# Intraoperative Music During General Anaesthesia in Dogs Undergoing Elective Ovariohysterectomy: A Prospective, Double-Blinded Randomized Exploratory Study

**DOI:** 10.3390/ani16010029

**Published:** 2025-12-22

**Authors:** Stefanos G. Georgiou, Pagona G. Gouletsou, Eleftheria Dermisiadou, Tilemachos L. Anagnostou, Aikaterini I. Sideri, Apostolos D. Galatos

**Affiliations:** 1 Clinic of Surgery, Faculty of Veterinary Science, School of Health Sciences, University of Thessaly, 43100 Karditsa, Greece; eldermis@vet.uth.gr (E.D.); ksideri@vet.uth.gr (A.I.S.); agalatos@vet.uth.gr (A.D.G.); 2 Clinic of Obstetrics and Reproduction, Faculty of Veterinary Science, School of Health Sciences, University of Thessaly, 43100 Karditsa, Greece; ngoule@vet.uth.gr; 3 Anaesthesia and Intensive Care Unit, Faculty of Health Sciences, School of Veterinary Medicine, Aristotle University of Thessaloniki, 54627 Thessaloniki, Greece; tanagnos@vet.auth.gr

**Keywords:** dog, music, general anaesthesia, analgesia, perioperative period, ovariohysterectomy

## Abstract

Music is considered a non-pharmacological adjunct in human anaesthesia, contributing to anaesthetic- and analgesic-sparing effects, modulating autonomic responses, and enhancing recovery. However, its effects in veterinary surgical settings remain largely unexplored. This study aimed to explore the potential influence of intraoperative music on anaesthetic and analgesic requirements, autonomic parameters, intraoperative adverse effects, and recovery quality in dogs undergoing elective ovariohysterectomy under general anaesthesia. In this prospective, randomized exploratory study, client-owned female dogs (*n* = 28) were randomly assigned to either a music group (exposed to instrumental classical music intraoperatively) or a control group (no music). All dogs received a standardized anaesthetic protocol. Mean end-tidal isoflurane concentrations, intraoperative analgesic requirements, heart rate, respiratory rate, blood pressure, adverse effects, and recovery quality were recorded and compared between groups using unpaired *t*-test, Mann–Whitney *U* test, or Fisher’s exact test, as appropriate (*p* < 0.05). No statistically significant differences were observed. Therefore, intraoperative music did not produce measurable effects on the assessed parameters. While no apparent benefit was observed in this study, future studies with larger sample sizes should investigate music-based interventions in more challenging or variable clinical scenarios. Additionally, further research is needed to clarify the extent to which anaesthetics suppress auditory processing. This exploratory investigation contributes to the limited body of evidence on auditory stimulation in veterinary anaesthesia.

## Simple Summary

Music has been increasingly explored as a supplementary tool in human medicine to improve postoperative outcomes in various surgical procedures. In veterinary medicine, the use of music as a perioperative intervention remains relatively under-investigated. Our work aims to explore whether the incorporation of music during the intraoperative period influences anaesthetic and analgesic parameters in 28 healthy dogs undergoing ovariohysterectomy under general anaesthesia. The dogs were divided into two groups of 14, and each group was exposed intraoperatively to either classical music or silence. All dogs received a standardized anaesthetic protocol. We observed that music had no apparent effect on any of the parameters evaluated. Further investigation of music as a supportive tool during the perioperative period may help clarify its potential clinical and welfare relevance in companion animals.

## 1. Introduction

Ovariohysterectomy (OVH) is a common and routinely performed surgical procedure in small animal practice [1] and was reported to be one of the most common surgical procedures performed in dogs in the United States of America [2]. It seems to be a relatively standardized source of soft tissue pain [2] and has been used as a model for the assessment of visceral pain and evaluation of the efficacy of the analgesic regimen [2,3], because the anticipated postoperative pain is predictable, modifying postoperative behavior for up to 24 h [4,5]. Abdominal surgeries such as spay contribute to ovarian stimulation pain, causing undesired autonomic responses in small animals [3]. According to published studies, stimulation of the ovary and ovarian ligaments, because of traction and ligature of the ovarian pedicles in dogs, is an adequate and repeatable means of producing visceral stimulation and is recognized as the most painful stimuli during OVH, requiring rescue analgesia in dogs [3]. More specifically, the laparotomy during OVH does not increase blood pressure (BP); however, clamping, ligating, and transecting the ovarian pedicle causes the greatest increase in BP during OVH (associated with signs of noxious stimuli) and are therefore considered to be the maximal surgical trauma and source of nociception during OVH [1,6]. Recent guidelines strongly propose the use of general anaesthesia and multimodal analgesia regimens for the management of postoperative pain in dogs undergoing OVH [5]. Furthermore, according to relative reports, the combination of an opioid and a non-steroidal anti-inflammatory agent (NSAID) contributes to an adequate level of postoperative analgesia in dogs undergoing OVH [5,7], while analgesics should be administered to dogs for at least 3 days postoperatively [5].

Music therapy has been proposed as a supportive approach in a variety of clinical conditions in humans, including hypertension [8], epilepsy [9,10], anxiety, depression, dementia, Parkinson’s disease [11], acute and chronic pain [12,13], and cancer [11,14], either as an adjunct to conventional procedures and pharmacotherapies or by itself. In accordance with human studies, music interventions have been considered as a method of environmental (auditory) enrichment in animals, as well. Music has been proposed to enhance animal welfare by decreasing anxiety, stress, or aggressive behaviours when physiological or behavioural parameters are being assessed [15,16]. More specifically, it has been reported that music elicits favourable physiological responses indicative of parasympathetic nervous system dominance in dogs [17,18] and promotes behaviours associated with reduced stress levels in both dogs [17,18,19,20,21,22,23,24] and cats [25,26,27].

Music’s effect on the perioperative period has also been evaluated, with existing evidence supporting its incorporation into the perioperative setting in human medicine as an adjunctive tool to improve postoperative outcomes in various surgical procedures [28,29,30,31,32,33,34]. However, the available literature on companion animals remains limited and is currently restricted to four studies conducted in dogs [35,36,37,38] and two studies in cats [39,40]. Favourable responses were reported when dogs were exposed to music preoperatively [36], intraoperatively [38], and postoperatively [37]; however, another study found no effect from music specifically designed for dogs on the level of dexmedetomidine-induced sedation [35]. Regarding the studies in dogs that have reported favourable outcomes, music interventions have contributed to increased levels of sedation preoperatively [36], anaesthetic- [36,38] and analgesic-sparing effects [37,38], and lower postoperative anxiety levels [37]. Additionally, cats under anaesthesia have exhibited significantly lower heart rate (HR), respiratory rate (RR), and systolic BP values when exposed to music compared to silence [39,40].

It has been proposed that music may have an effect even when applied intraoperatively, under general anaesthesia; however, the potential effects of music in patients under general anaesthesia still remain an area of contention among researchers, both in human and veterinary medicine. Reviews and meta-analyses regarding the effect of intraoperative music interventions under general anaesthesia have revealed significant pain- and anxiety-reducing effects in humans [29,31,41], correlating with lower opioid consumption and increased patient satisfaction [30,41]. Notably, the recent systematic review and meta-analysis of Fu et al. [41] showed that these effects were specifically related to music interventions but not related to intraoperative therapeutic suggestions, which had no apparent effect on patients’ recovery. Similar observations have been recorded in companion animals, though to a lesser extent. There has only been one study evaluating the effect of music on anaesthetized dogs [38] and two studies on anaesthetized cats [39,40]. Dogs under a light-anaesthetic plane undergoing minor skin surgery exhibited anaesthetic- and analgesic-sparing effects, according to the intraoperative isoflurane and fentanyl requirements assessment, when exposed to classical music intraoperatively compared to a silent condition [38]. However, no significant effect of intraoperative music was observed on physiological parameters (HR, RR, and BP). On the contrary, the two existing studies in cats presented for elective OVH reported lower HR, RR, and systolic BP values when exposed to classical music under general anaesthesia compared to both other music genres (pop and heavy metal music) and a silent scenario [39,40].

Although there is evidence supporting auditory information processing under deep sedation or even under general anaesthesia in humans [41,42,43,44], as well as indirect evidence in companion animals [38,39,40], general anaesthetics appear to suppress the brain’s capacity to process sensory information in a dose-dependent manner [42,45,46,47,48]. More specifically, this has been directly demonstrated for general anaesthetics such as propofol [42,43,44,48,49], isoflurane [45,49], sevoflurane [47,50], and ketamine [46,48], using auditory evoked potentials [45,46,47], electroencephalogram-based approaches [44,49], or functional magnetic resonance imaging [42,43,48,50]. The ability of auditory stimuli to be perceived and partially processed during general anaesthesia—particularly on lighter anaesthetic planes—has been primarily investigated in the context of assessing the potential for intraoperative awareness or auditory memory formation to ensure adequate suppression of consciousness and maintaining sufficient anaesthetic depth. In summary, while brainstem-level responses appear to be preserved at light levels of anaesthesia, progressive disruption of cortical and auditory processing, as well as memory function, is observed at higher anaesthetic doses [42,43,44,45,46,47,48,49,50]. In line with these findings, although intraoperative music has been associated with beneficial effects in patients under general anaesthesia, these effects appear to be less pronounced compared to those observed in conscious patients [29].

The objective of this exploratory, prospective randomized study was to explore the influence of intraoperative music on anaesthetic and analgesic requirements, autonomic responses, incidences of intraoperative adverse effects, and recovery quality in dogs undergoing elective OVH under general anaesthesia. Given the limited existing literature on this topic in veterinary anaesthesia, a prospective, randomized exploratory design was chosen to assess whether meaningful trends or associations could be identified. Rather than testing a specific hypothesis, this investigation aimed to provide preliminary insights that may inform future, more targeted research on the role of music in the perioperative management of veterinary patients.

## 2. Materials and Methods

### 2.1. Study Design

This was a prospective, double-blinded, randomized, clinical study that was designed to investigate the effect of intraoperative music during general anaesthesia in dogs undergoing elective OVH. The study was conducted at the surgery clinic of the Faculty of Veterinary Science of University of Thessaly. It was approved by and followed the guidelines of the Animal Ethics Committee of Greece (license number 1117/45296, 21 March 2017) regarding animal experimentation. All methods were performed in accordance with relevant guidelines and regulations, and the authors complied with the ARRIVE guidelines for in vivo animal research and the CONSORT statement for randomized controlled trials. A written informed owner’s consent was obtained before each dog’s inclusion in the study.

### 2.2. Animals

A total of 28 adult, healthy, female, client-owned dogs scheduled to undergo routine OVH surgery were used in this study. Dogs with normal auditory function, properly vaccinated, and deemed clinically healthy (ASA 1), based on physical examination and haematological and biochemical profile, were included in the study. Furthermore, only dogs that had no reported previous exposure to music as part of their daily routine, according to their owners, were included in the study. Auditory function was assessed by history, otoscopic examination of the external ear and tympanum radiography of the tympanic bullae, neurologic examination, and clinical observation of the response to a sound stimulus outside the animal’s visual field, as has already been proposed in a relevant study [38]. This was previously evaluated in a former appointment, at least a month earlier. Dogs < 6 months or >8 years old, <5 kg or >30 kg, obese, lactating, pregnant, fearful, or aggressive were excluded from the study. Further exclusion criteria were prior abdominal surgery; pre-existence of any acute or chronic pain conditions; and administration of opioids, NSAIDs, or corticosteroids in the previous 60 days.

### 2.3. Randomization and Blinding

Dogs that fulfilled the eligibility criteria and their owner provided written informed consent were randomly allocated to either the control group (NM), where no music was played intraoperatively, or to the music intervention group (MM), where they were exposed to classical music during the intraoperative period, using an online randomization tool (research randomizer, http://www.randomizer.org, accessed on 21 December 2025). If a dog was excluded during the study, the next one was allocated to the same treatment. Computerized randomization revealed the allocated intervention to a particular member of the research team, being the only individual aware of the treatment group allocation, who was responsible for applying the headphones to the dogs and preparing the respective intraoperative intervention (music or silence). The current, nonblinded researcher was also responsible for removing the headphones from each dog’s ears when the surgery was completed but was not further involved in the procedure. As all included dogs were operated on under general anaesthesia and wore headphones covering the whole ear during the intraoperative period, both the dogs and the clinicians involved in the perioperative care (anaesthetist and surgeon) were unaware of the type of intervention, ensuring the double-blind state of the study. Furthermore, the statistician who performed the statistical analysis of the data was blinded to the study design, as well. De-blinding was only performed after finalization of the data analysis.

### 2.4. Music Intervention

Dogs in the MM group were exposed to Mozart music (Sonata for two pianos in D-major, K.448, andante) only intraoperatively under general anaesthesia via headphones covering the whole ear (ATH-M50X, Audio-Technica Corporation, Tokyo, Japan). The second movement (andante) of Mozart’s sonata K.448 that was employed had a total duration of 10 min. It is a slow tempo movement of approximately 70 beats per minute (BPM) without abrupt dynamic alterations or abruptly shifting tones, and the instrumentation is rather simple, consisting solely of 2 pianos. The volume of the stimulus was set approximately at 60 dB, as confirmed by a decibel meter, and remained stable throughout surgery. The respective musical piece was played repeatedly on a loop for the whole duration of the surgery. The dogs in the control group (NM) wore the same headphones covering the whole ear, just as the dogs in the music intervention group; however, no music was played.

After successful intubation of the trachea and proper positioning of the dog, the headphones were placed on all dogs’ ears, and music was initiated for the MM group. They remained in place throughout the whole surgery and were eventually removed at the end of anaesthesia.

### 2.5. Procedure (Preoperative Period, Anaesthesia, and Surgery)

Dogs were admitted to the university facilities the morning before surgery, at least 2 h before the procedure initiation, for preoperative evaluation, bloodwork, and to be acclimatized to the environment and personnel. After clinical evaluation and blood sampling, the dogs remained in a quiet environment until premedication administration, while being allowed to investigate the preparation room and interact with a member of the research team. Dogs’ owners were advised to comply with an 8-h preoperative fasting, while water was withheld for 2 h. After the acclimatization period, dogs were premedicated with 0.05 mg/kg acepromazine (Acepromazine, Alfasan, Woerden, The Netherlands) and 20 μg/kg buprenorphine (Bupaq, Neocell, Athens, Greece) intramuscularly (IM). Approximately 30 min later, the cephalic vein was catheterized aseptically, and lactated Ringer’s solution was administered at a rate of 5 mL/kg/h. Oxygen at 4 L/min was supplied via a face mask for 3 min before and during induction of anaesthesia with intravenous (IV) propofol (Propofol, Fresenius Kabi, Athens, Greece) increments of 1 mg/kg until tracheal intubation. The endotracheal tube was connected to a circle anaesthetic circuit, and maintenance of anaesthesia was performed with a mixture of isoflurane (Isoflo, Abbott, Maidenhead, UK) in 100% oxygen. Afterwards, 20 mg/kg of cefuroxime (Zinacef, GlaxoSmithKline, Athens, Greece) was administered IV, 0.1 mg/kg of meloxicam (Metacam, Boehringer Ingelheim, Ingelheim am Rein, Germany) was administered subcutaneously (SC), the headphones were adjusted properly, and the dog was prepared for surgery. SpO_2_; HR; RR; non-invasive arterial (systolic, mean, and diastolic) BP; partial pressure of expired carbon dioxide (PECO_2_); inspired and expired isoflurane concentrations; and rectal temperature were recorded intraoperatively every 5 min until the end of anaesthesia using a multiparametric monitor (Datex Ohmeda S/5, Datex-Ohmeda Division, Instrumentarium Corp, Helsinki, Finland). The temperature was strictly maintained between 37 °C and 38 °C by means of a forced-air heating system whenever needed, and spontaneous ventilation was permitted. A routine midline OVH was performed by the same experienced veterinary surgeon every time, and surgery was initiated only when each dog had achieved the desired anaesthetic depth (absence of palpebral reflex and jaw tone). The isoflurane vaporizer dial was adjusted accordingly, targeting an end-tidal isoflurane concentration of 1.3% for at least 5–10 min before surgery initiation, which corresponds to the MAC for isoflurane as has been determined in dogs [51]. During anaesthesia, the desired anaesthetic depth was based on neurological assessment, while acute nervous system responses (acute or abrupt increases in HR, BP, and RR) associated with surgical stimulation were used to monitor the adequacy of analgesia. If the neurological assessment indicated a decrease in the anaesthetic depth, the isoflurane concentration increased by 0.2%. In case of a dramatic anaesthetic depth decrease, 1 mg/kg IV propofol was administered, and the dog was excluded from the study because of unacceptable anaesthetic depth fluctuations compromising the study design uniformity. Any increase in physiologic parameters (HR, BP, and RR) > 20% from baseline associated with surgical stimulation was considered indicative of inadequate analgesia, and 1 μg/kg IV fentanyl (fentanyl, Janssen–Cilag, Athens, Greece) was provided as rescue analgesia. In either case, application of the nociceptive stimulus ceased and was not re-applied until the altered parameter returned to baseline values. After a delay of 10 min, all parameters were reassessed, and if all values were at baseline levels again, the procedure was continued. If not, another fentanyl bolus of 1 μg/kg was re-administered, and a further delay of at least 10 min was allowed. If again that was not enough to ameliorate the respective ANS response, a constant rate infusion of fentanyl at 5–10 μg/kg/h was initiated, and the dog was excluded from the study due to analgesic protocol failure and an unacceptable extension of the surgical duration. Bradycardia or hypotension were defined as HR < 60 beats/minute or mean BP < 60 mmHg for more than 5 min. Bradycardia or hypotension incidents were treated with atropine at 0.02 mg/kg IV or dopamine at 7 μg/kg/min, respectively. Mechanical ventilation was initiated if PECO_2_ was >50 mmHg.

At the end of surgery, isoflurane was discontinued, and the dogs were placed in sternal recumbency until extubation. Afterwards, they were transported to a warm and silent recovery room, while continuously monitored, until full recovery. In case of an unacceptable recovery (rough recovery with vocalization or severe dysphoria), propofol at 0.5 mg/kg IV was administered. If that was not enough, another 0.5 mg/kg IV bolus of propofol was re-administered, along with buprenorphine at 10 μg/kg IV. If that was also ineffective, fentanyl at 5 μg/kg/h was initiated, and the dog was excluded from the study because of failure of the analgesic plan. If the recovery was uneventful, a buprenorphine dose of 10 μg/kg was administered IM just before the dogs’ discharge, unless it was required earlier during the immediate postoperative period as a rescue analgesia. The dogs were discharged from the hospital on the same day, when fully alert and conscious, and meloxicam at 0.1 mg/kg orally was continued once daily for the following 5 days.

### 2.6. Outcome Measures

The main objective of the current study was to investigate the potential beneficial effects of music on anaesthesia and analgesia in dogs submitted to OVH, when it is applied only intraoperatively, under general anaesthesia. The music’s effect on anaesthesia was assessed by evaluating the isoflurane requirements, while its effect on analgesia was assessed by evaluating the intraoperative analgesic requirements. Anaesthetic requirements for each group were evaluated by calculating the mean end-tidal isoflurane concentration of each dog during the intraoperative period, defined as the time from induction of anaesthesia until the end of anaesthetic maintenance. The effect of music on analgesia was evaluated by recording the prevalence of rescue analgesia during the intraoperative period and by calculating the mean fentanyl dose, which was administered as rescue analgesia in each dog, according to the allocated group.

Secondary outcome measures were the intraoperative physiologic variables, the incidence of perioperative complications (adverse effects), and the quality of recovery. The mean HR; RR; and the systolic, mean, and diastolic BP were calculated for each dog during the intraoperative period, according to the intervention (music or silence). Adverse effects during the intraoperative or immediate postoperative period such as bradycardia, arrhythmias, hypotension, or hypercapnia and the quality of recovery were also recorded, either related to the music intervention or not. Recovery quality was assessed as acceptable (ideal, smooth, and uncomplicated without vocalization) or unacceptable (difficult or rough with vocalization or dysphoria), according to a previous study [52].

Furthermore, anaesthesia and surgery duration were recorded. Anaesthesia duration was defined as the time from intubation until the discontinuation of isoflurane administration, while surgery duration was the time from the first (skin) incision until the last suture placement and skin wound closure.

### 2.7. Sample Size Justification

A formal sample size calculation was not conducted prior to the study initiation due to the limited availability of relevant literature and the absence of reliable estimates for expected effect sizes. The sample size of 14 dogs in each group (28 dogs in total) was therefore determined based on reasonable considerations and documented justifications for exploratory studies in novel research areas. Julius [53] recommended a minimum of 12 subjects per group to obtain reliable estimates of variability, and Whitehead et al. [54] supported similar sample sizes for research intended to inform future studies. Furthermore, similar studies in dogs and cats investigating the effect of music during the perioperative period have used comparable sample sizes ranging from 10 to 20 animals [35,36,37,38,39,40]. More specifically, previous studies investigating the effect of music during the intraoperative period have used sample sizes ranging from 12 [39,40] to 20 [38] subjects, suggesting that the current sample of 28 dogs is sufficient to identify meaningful trends and guide future research. Additionally, the study design (prospective, randomized, double-blinded clinical study) enhances statistical efficiency and helps mitigate the limitations of a smaller sample. This study intends to serve as a foundation for future research, including larger, hypothesis-driven, adequately powered studies.

### 2.8. Statistical Analysis

Data were analyzed using IBM SPSS Statistics 29. The normality of data distribution was assessed using the Shapiro–Wilk test. Normally distributed continuous variables are presented as the mean ± standard deviation (SD), while non-normally distributed variables are summarized as the median (range). Statistical significance was set at *p* < 0.05.

Comparisons between the music and control groups for normally distributed continuous variables, including mean end-tidal isoflurane concentrations, mean fentanyl dose, HR, RR, and BP, were performed using an unpaired *t*-test. Non-normally distributed variables, such as the duration of intraoperative hypotension, were compared using the Mann–Whitney *U* test. Age, weight, anaesthesia, and surgery duration were analyzed with a two-tailed unpaired *t*-test.

Categorical variables, including the number of dogs requiring rescue analgesia, exhibiting adverse effects, and recovery quality, were compared using Fisher’s exact test.

## 3. Results

### 3.1. Study Population

A total of 28 dogs were randomized into group MM (n = 14) and group NM (n = 14), according to the type of the intraoperative intervention (music vs. silence). The descriptive characteristics of this population are presented in Table 1. No dog was excluded from the study due to either a dramatic depth of anaesthesia decrease or due to analgesic protocol failure intraoperatively, as was defined.

### 3.2. End-Tidal Isoflurane Concentrations (%)

End-tidal isoflurane concentrations were recorded every 5 min intraoperatively for each of the 28 dogs. The mean end-tidal isoflurane concentration was calculated for each dog across the entire anaesthetic maintenance period, from induction until the end of anaesthesia, to provide an estimate of anaesthetic requirements for each group (MM vs. NM), according to the intraoperative intervention, as a measure of overall anaesthetic consumption.

The mean end-tidal isoflurane concentration did not differ significantly between dogs in the MM and NM groups (Table 1).

### 3.3. Fentanyl Requirements (μg/kg)

Rescue analgesia was administered to 3/14 dogs (21.4%) in the MM group and 5/14 dogs (35.7%) in the NM group. The prevalence of rescue analgesia was not significantly different between treatments (*p* = 0.41) (Table 2). In both groups, one dog required more than one rescue fentanyl bolus (two boluses in total).

Analgesic requirements were evaluated by recording the total intraoperative fentanyl dose administered to each dog, according to the intraoperative intervention. The mean values of the fentanyl requirements were not significantly different between the MM and the NM groups (Table 1).

### 3.4. Physiologic Variables (HR, RR, Systolic BP, Mean BP, and Diastolic BP)

Physiologic values were recorded every 5 min during the intraoperative period, and the mean value was calculated for each parameter per animal per group. No statistically significant differences in HR; RR; or BP (systolic, mean, and diastolic BP) were observed between the music (MM) and the no music (NM) treatments during the intraoperative period (Table 3).

### 3.5. Adverse Effects (Bradycardia, Arrhythmias, Hypotension, and Hypercapnia)

The predetermined adverse effects were observed in 4/14 (28.5%) dogs in the MM group, while 5/14 (35.7%) dogs exhibited the respective adverse effects in the NM group (Table 2); however, the difference was not statistically significant (*p* = 0.68).

No dog exhibited bradycardia (HR < 60 beats/min) or arrhythmias. Hypercapnia was observed in 2/14 (14.3%) dogs in the MM group and in 2/14 (14.3%) dogs in the NM group.

Intraoperative hypotension (mean BP < 60 mmHg for more than 5 min) was observed in 2/14 (14.3%) dogs in the MM group and 3/14 (21.4%) in the NM group, with no significant difference between groups. The average duration of hypotension was 0 (0–15) minutes in the MM group and 0 (0–15) minutes in the NM group and did not differ between treatments (Mann–Whitney *U* = 104.5, *p* = 0.769). All hypotensive episodes were rapidly corrected following the initiation of dopamine. No other adverse effects were recorded.

### 3.6. Recovery Quality

No difference was observed in terms of recovery quality between groups MM and NM, as two dogs (14.2%) in each group exhibited signs of unacceptable recovery (vocalization and dysphoria) (Table 2). No dog was excluded from the study, as they were all responsive to the propofol bolus of 0.5 mg/kg IV, as was predetermined.

## 4. Discussion

This exploratory, prospective, double-blind, randomized clinical study investigated the effects of a preselected, instrumental, classical music intervention applied solely during the intraoperative period using headphones covering the whole ear, compared to silence, on parameters related to anaesthesia and analgesia in dogs undergoing elective OVH under general anaesthesia. Specifically, we evaluated music’s effect on anaesthesia by recording intraoperative isoflurane requirements and on analgesia according to the intraoperative fentanyl requirements. We also investigated intraoperative autonomic responses, the quality of anaesthesia by recording any potential adverse effects, and the quality of recovery.

Rescue fentanyl was employed to ensure adequate intraoperative analgesia despite buprenorphine premedication, as buprenorphine alone may not sufficiently suppress autonomic responses during highly noxious phases of OVH, particularly traction, ligation, and transection of the ovarian pedicles, which represent the most painful components of the surgery [1,3,6]. The 10-min delay after IV fentanyl administration provided a conservative margin to ensure maximal analgesic effect and stabilization of the physiologic parameters (HR, BP, and RR), with return to baseline values before continuation of the procedure. This approach minimized the risk of residual nociceptive responses that could confound intraoperative measurements and ensured a consistent assessment of analgesic adequacy. Furthermore, propofol was used to manage unacceptable or dysphoric recovery due to its rapid onset of action, short duration, and predictable effect after IV administration, consistent with previous research in which recovery quality was assessed using the classification system described by Bustamante et al. [52], which was also applied in the present study. However, no statistically significant favourable effects of the music intervention were identified on any of the parameters investigated.

Music has been proposed to exert beneficial effects on various perioperative outcomes in humans across different surgical populations and procedures [28,29,30,31,32,33,34,41]. Similar findings have been reported in dogs and cats, with music associated with favourable effects when applied preoperatively [36], intraoperatively [38,39,40], and postoperatively [37], although evidence in veterinary medicine remains limited. Systematic reviews and meta-analyses have demonstrated beneficial effects of music on pain, anxiety, and postoperative recovery in humans, even when applied intraoperatively under general anaesthesia, albeit with less pronounced effects compared to conscious patients [29,30,31,41]. In veterinary medicine, this topic remains largely unexplored, with only three published studies evaluating music during general anaesthesia: two in cats [39,40] and one in dogs [38], all reporting favourable outcomes. In the present study, music was applied solely during general anaesthesia; however, no significant effects were observed for the parameters investigated. Several factors could account for the discrepancy between our findings and previous reports.

Firstly, the targeted surgical depth of anaesthesia, which was directed by both neurological assessment and the reported MAC in dogs [51], could have influenced our results. It is known that general anaesthetics such as propofol, isoflurane, sevoflurane, or ketamine suppress auditory function by reducing the brain’s ability to process and transmit auditory information, mainly in a dose-dependent manner [42,43,44,45,46,47,48,49,50]. That could be a reason why we observed no effect of intraoperative music in our study, in contrast to dogs in the study by Georgiou et al. [38], where music was also applied under anaesthesia and an anaesthetic- and analgesic-sparing effect of music was demonstrated. However, in the study by Georgiou et al. [38], a light-anaesthetic plane was targeted (present but reduced palpebral reflex and mild jaw tone), potentially preserving auditory processing capability to some extent, while, in the current study, we aimed for a surgical depth of anaesthesia (absence of palpebral reflex and absence of jaw tone), affecting auditory function more potently. More specifically, the study by Georgiou et al. [38] used mean end-tidal isoflurane concentrations of 1.0–1.1% to achieve the desired anaesthetic depth for the music groups, while, in the current study, a higher mean concentration of 1.38% was used to achieve the targeted surgical anaesthetic depth in the MM group. On the contrary, two studies in cats undergoing OVH proposed reduced anaesthetic isoflurane requirements and lower mean RR, HR, and systolic BP values when music interventions were used solely intraoperatively, concluding that cats are likely to preserve auditory sensory stimuli processing under general anaesthesia [39,40]. However, we cannot directly compare these results with the results of our study, as the studies by Mira et al. [39,40] did not mention the exact isoflurane concentrations used or the method of anaesthetic depth assessment. The fact that intraoperative music interventions are beneficial but seem to be affected by the level of consciousness or the depth of anaesthesia has been demonstrated in systematic reviews [29,31] and randomized controlled trials (RCTs) [55,56,57,58,59,60] in humans, as well. As was illustrated in our study, no effect of intraoperative music on inhalant [55,57], propofol [59], and opioid requirements [55,57,59] or on physiologic parameters [55,57], stress hormone levels [55,59], adverse effects during surgery [55,57,59], or quality of recovery [59] has been reported when patients were under BIS-guided general anaesthesia (i.e., BIS values 50–60). However, when the anaesthetic depth was lighter (BIS ≈ 70), the total consumption of anaesthetics was proven to be lower [56,58]. That was also confirmed in the systematic review by Hole et al. [29], who proposed that music’s impact was greater when patients were conscious compared to being under general anaesthesia.

Secondly, we chose to apply music solely intraoperatively instead of incorporating it during the whole perioperative period (from the preoperative to the postoperative period). Most of the existing studies in dogs and cats evaluated the effect of single-phase music interventions [35,36,37,39,40], either solely preoperatively [35,36] or postoperatively [37] in dogs or solely intraoperatively in cats [39,40]. The only study that used music in more than one phase of the perioperative period was the study by Georgiou et al. [38], where dogs were exposed to music both in the preoperative and intraoperative periods. This fact, along with the differences in the targeted depth of anaesthesia, could have played a major role in the observed discrepancy between the results of this study, which demonstrated anaesthetic- and analgesic-sparing effects of music, and the results of our study, where no such effect was observed. Furthermore, our results do not correlate with the results of Mira et al. [39,40], which described music-related beneficial effects in cats when music was applied solely intraoperatively, as in our study. Considering that the number of existing studies in dogs and cats is rather limited, it seems difficult to draw definite conclusions about the optimal timing of a music intervention. Literature evidence in humans has proposed that the timing of music delivery makes a minor difference in the study outcomes [29,31]. However, sub-group analyses in these systematic reviews found a greater effect on pain when music was played preoperatively compared to intraoperatively [29] or postoperatively compared to intraoperatively [31]. That could potentially further explain why we found no effect from solely intraoperative music in the current study compared to the study of Georgiou et al. [38], where music was offered both preoperatively and postoperatively. In addition to that, Hole et al. [29] proposed that a more profound music’s effect is exhibited in conscious compared to anaesthetized patients, while Poulsen and Coto [61] further recommended that music should be delivered throughout the entirety of the surgical procedure for the maximum effect. That was also proposed by a recent review manuscript that aimed to provide recommendations for the application of music perioperatively in dogs and cats [62].

Furthermore, we only evaluated the effect of music on parameters related to the intraoperative period, without investigating its potential effect on the forthcoming postoperative period. Although there is no existing data about the effect of intraoperative music on the postoperative period in veterinary literature, when music was delivered intraoperatively under general anaesthesia in human patients, its effect was advantageous on a variety of outcome measures, such as postoperative pain scores [63,64,65,66] and analgesic requirements [67], postoperative anxiety, depression and pain catastrophizing [67], quality of recovery [63,65,66], and adverse effects incidents [66]. Such effects were further confirmed by a more recent systematic review and meta-analysis where intraoperative music during general anaesthesia reported a significant moderate-to-large beneficial effect on postoperative pain and opioid requirements for the first 24 h after surgery [41]. Therefore, if we further investigated the role of intraoperative music in the postoperative period, instead of solely intraoperatively, we could potentially observe some benefits. Although most systematic reviews and meta-analyses seem to support intraoperative music interventions, even under general anaesthesia [29,31,41], some both older and more recent RCTs found no benefit from intraoperative music on postoperative pain (neither pain scores nor opioid requirements) [55,57,59,60], intraoperative anaesthetic and analgesic medications [55,57,59], stress response to surgery [55,59], intraoperative autonomic responses [55,57], or quality of recovery and adverse effects [55,57,59], partly correlating with the results of our study, where we also observed no favourable effect of intraoperative music on a variety of outcome measures.

Another reason that could potentially explain the discrepancy between the results of our study and those of the rest of the veterinary studies incorporating music into the intraoperative period [38,39,40] could be the choice of the music intervention itself. It has been proposed that parameters such as the type of music (genre, tempo, instrumentation, pitch, and volume); the timing of the intervention (solely preoperatively, intraoperatively, or postoperatively, or at multiple perioperative timepoints); the individual who selects the music; the duration of the music intervention; and the participant’s history and listening habits seem to be important components to be considered when designing a perioperative music therapy intervention, according to systematic reviews and reported guidelines in human patients [29,30,31,32,33,61,68]. That has also been proposed for perioperative music interventions in dogs and cats [16,62]. Although we carefully chose the type of music and took into consideration the features of the music intervention according to the existing human [29,30,31,32,33,61,68] and veterinary literature [38,39,40,62], we found no effect from music’s application intraoperatively under general anaesthesia on any of the parameters evaluated. In our study, we used a classical Mozart music excerpt delivered with headphones covering the whole ear, with specific characteristics according to tempo, instrumentation, pitch, and volume, as has been described above (Section 2.4). The particular music genre (classical music), although it seems to be a rather subjective human definition, was found to be the best choice for dogs’ and cats’ perioperative period [36,38,39,40], even when applied intraoperatively [38,39,40], and slow-tempo, simple-orchestrated tracks not exceeding a volume of 65 dB have been previously used in similar settings contributing to desirable effects [38]. More specifically, we used exactly the same music track that contributed to anaesthetic- and analgesic-sparing effects in the studies by Georgiou et al. [36,38]: the second movement of Mozart’s sonata K.448 is a piano-based, slow-tempo movement of approximately 70 BPM, and the volume was set at a level of 60 dB. Classical music with a volume < 80 dB was also related to the most desirable responses under general anaesthesia in cats compared to the control conditions, pop and heavy metal music [39,40]. However, the authors of these studies did not mention the exact tempo of the classical music piece, and the instrumentation was not piano-based but string-based instead. Therefore, considering that the respective music features were comparable between our study and the other veterinary studies, we can assume that the discrepancy in these results cannot be strongly attributed to the music intervention itself but potentially to either some of the reasons mentioned above (i.e., surgical depth of anaesthesia or the timing of intervention) or to other parameters irrelevant to the features of the music intervention itself.

Going further with the investigation of the factors that contributed to the lack of any observed effect of intraoperative music in our study, we should consider one more parameter that could potentially have played a role. In our study, we only included dogs that, according to their owners, had no reported previous exposure to music as part of their daily routine. Most of the studies in dogs and cats mention no information about previous exposure to music, either when applied as an environmental enrichment strategy [17,18,19,20,21,22,25,26,27,69] or during the perioperative period [37,39,40]. However, a recent study investigating the music preference or the effect of music familiarity in dogs found that music-naive dogs exhibited no clear preference for classical music, while dogs that were routinely exposed to classical music at home exhibited more relaxed behaviours when exposed to the same type of music [24]. This observation reveals that the potential pet’s familiarity with certain music compositions could be of importance, further corresponding to human literature evidence, as the participant’s history and listening habits seem to be important components to be considered when designing a perioperative music therapy intervention, according to systematic reviews in human patients [29,30,31,34]. In line with these findings, recent recommendations aiming to guide a stepwise approach for perioperative music interventions in dogs and cats propose that previous music exposure or the potential animal’s familiarity with music should be considered when designing a perioperative music intervention [62]; however, other factors should be considered, as well. Additionally, two recent studies in dogs found that, although the included canine subjects were not familiar with music listening (as part of their daily routine), their exposure to music preoperatively [36] or intraoperatively [38] resulted in favourable outcomes.

Finally, another reason that could have contributed to the lack of any observed effect of intraoperative music in our study could be the level of perioperative analgesia provided. OVH has been proposed to contribute to moderate postoperative pain [5], and buprenorphine combined with an NSAID has been recommended for postoperative analgesia in such surgical operations in dogs [5,7]. Considering that music, as a non-pharmacological intervention, has been proposed to exert only a moderate effect on conscious dogs [23], we can assume that the potent perioperative analgesic protocol used (buprenorphine and meloxicam) masked the potential effect of music during the intraoperative period, if any. That could explain both the low proportion of patients that required rescue analgesia intraoperatively or exhibited signs of unacceptable recovery quality in both groups and the insignificant differences regarding all the parameters investigated between the MM and the NM groups. Another finding that could support the fact that music’s effect as a non-pharmacological intervention is mild could be the lack of any observed effect of music in dogs premedicated with the potent sedative dexmedetomidine [35] compared to a mild premedication protocol consisting of acepromazine and butorphanol where music contributed to a depth of sedation increase and a propofol-sparing effect [36].

This study has several (methodological) strengths regarding its design. The dogs, the clinicians involved in the perioperative care (anaesthetist and surgeon), and the staff handling the data (statistician) were all blinded to the intervention and unaware of patient allocation to ensure that the research team could not influence the study results. Randomization was employed to assign dogs to study groups in order to minimize selection bias, and as a result, any differences observed could be attributed to the intervention itself rather than to potential pre-existing differences between groups. A further strength of the present study was the prespecified surgical depth of anaesthesia and the strict anaesthetic depth assessment methods, which ensured that all dogs were under the same anaesthetic depth during surgery. Additionally, the components of the music intervention were carefully considered, according to propositions originating from veterinary [62] and human literature [29,30,31,32,33,61,68], which state that the unclear description of the characteristics of music interventions is a limitation in veterinary and human studies, contributing to heterogeneity. Finally, we incorporated music solely into the intraoperative period so that the conclusions could be attributed exclusively to the respective period and not to more phases of the perioperative period, which could further complicate the extraction of proper conclusions.

However, this study also has some limitations. Firstly, the approximately 2-h acclimatization period might not have been enough. It has been illustrated that veterinary clinics are considered stressful and challenging environments for dogs and cats, leading to high levels of stress and negatively affecting their welfare [22,69,70,71], with those consequences potentially being reflected in the perioperative period as well. However, the fact that client-owned dogs were included in the study made it difficult to employ longer acclimatization periods. Secondly, a key limitation of this exploratory study was the reliance on mean values for the end-tidal isoflurane concentration; HR; RR; and systolic, mean, and diastolic BP to assess the anaesthetic depth and physiological responses rather than analyzing these parameters at multiple intraoperative timepoints. This approach was intentionally selected to align with the primary aim of the study, which was to explore the effects of intraoperative music on parameters related to anaesthesia and analgesia between music-exposed and control groups under a stable surgical anaesthetic plane. Given the relatively short duration and stable nature of the surgical procedures (≈45 min), significant temporal fluctuations in these variables were not expected. Thus, the use of mean values was considered a practical, representative, and clinically relevant method for capturing overall trends, minimizing unnecessary data complexity, and supporting the preliminary hypothesis-generating nature of the investigation. Additionally, frequent measurements (every 5 min) were performed to ensure accuracy, and any major deviations or events were documented (i.e., incidence of adverse effects) or treated (i.e., fentanyl requirements recording) separately, if they occurred. Finally, although the relatively small sample size represented a limitation, the prospective, randomized, double-blinded study design enhanced the internal validity and helped mitigate this constraint. Given the limited existing literature and the absence of reliable estimates for expected effect sizes, a formal a priori power analysis was not feasible. Therefore, the sample size of 28 dogs (14 per group) was determined based on feasibility considerations, consistent with the recommendations of Julius [53], who noted that small sample sizes may be appropriate for early-phase or exploratory clinical research. As an exploratory investigation, the present study was not designed to detect predefined effect sizes but, rather, to generate preliminary observations that may guide the design and sample size justification of future hypothesis-driven, adequately powered studies.

## 5. Conclusions

No beneficial effect of intraoperative music was observed in dogs undergoing elective OVH under general anaesthesia. Slow-tempo, piano-based, classical instrumental music played during surgery did not provide an anaesthetic- or analgesic-sparing effect, likely due to the surgical depth of anaesthesia or sufficient perioperative analgesia, which may have masked any mild effect of music as a non-pharmacological adjunct. Future adequately powered, larger-scale studies should evaluate the impact of non-pharmacological interventions like music during the perioperative, or specifically the intraoperative, period in dogs, particularly in longer or more painful procedures (e.g., orthopaedic surgeries) or high-risk patients. It would also be valuable to investigate the extent to which general anaesthetics suppress auditory function.

## Figures and Tables

**Table 1:** Age, weight, duration of anaesthesia, duration of surgery, end-tidal isoflurane concentration, and fentanyl requirements for 28 dogs undergoing OVH while being exposed to either Mozart music (MM) or no music (NM) intraoperatively under general anaesthesia. No significant differences were found with regard to age, weight, duration of anaesthesia, and duration of surgery between groups. Data are reported as mean ± SD (standard deviation).

Data	MM (*n* = 14)	NM (*n* = 14)	*p*-Value
Age (years)	3.4 ± 1.9	2.6 ± 1.2	0.23
Weight (kg)	13.4 ± 7.5	17.5 ± 8.1	0.21
Anaesthesia duration (minutes)	55.7 ± 4.1	57.2 ± 3.5	0.35
Surgery duration (minutes)	43.7 ± 4.5	45.2 ± 3.1	0.36
End-tidal isoflurane concentration (%)	1.38 ± 0.07	1.42 ± 0.08	0.21
Fentanyl requirements (μg/kg)	0.4 ± 0.8	0.7 ± 0.9	0.38

**Table 2:** Prevalence of intraoperative rescue analgesia, adverse effects, and recovery quality in dogs exposed to Mozart music (MM) or no music (NM) intraoperatively. No significant differences were observed regarding these parameters between groups. Rescue analgesia and adverse effects were recorded as yes/no. Recovery quality was classified as acceptable/unacceptable.

Parameter	Classification	MM (*n* = 14)		NM (*n* = 14)	
		*n*	%	*n*	%
Intraoperative prevalence of rescue analgesia	No	11	78.6%	9	64.3%
	Yes	3	21.4%	5	35.7%
Adverse effects	No	10	71.5%	9	64.3%
	Yes	4	28.5%	5	35.7%
Recovery quality	Unacceptable	2	14.2%	2	14.2%
	Acceptable	12	85.8%	12	85.8%

**Table 3:** Heart rate (beats/min); respiratory rate (breaths/min); and systolic, mean, and diastolic blood pressure (mmHg) in dogs exposed to Mozart music (MM) and in dogs not exposed to music (NM) intraoperatively under general anaesthesia. No significant differences were found with regard to the respective physiologic variables between groups. Data are reported as mean ± SD.

Physiologic Variables	MM (*n* = 14)	NM (*n* = 14)	*p*-Value
Heart Rate (beats/min)	113.7 ± 15.9	122.3 ± 14.7	0.18
Respiratory Rate (breaths/min)	10.9 ± 1.9	11.1 ± 1.7	0.78
Systolic Blood Pressure (mmHg)	103 ± 12.1	105 ± 14.5	0.72
Mean Blood pressure (mmHg)	69.3 ± 6.8	67.7 ± 7.2	0.58
Diastolic Blood Pressure (mmHg)	51.2 ± 6.2	50 ± 8.6	0.71

## Data Availability

The data presented in this study are available on request from the corresponding author.
